# Ultra-high-field MR in Prostate cancer: Feasibility and Potential

**DOI:** 10.1007/s10334-022-01013-7

**Published:** 2022-05-17

**Authors:** Carlijn J. A. Tenbergen, Gregory J. Metzger, Tom W. J. Scheenen

**Affiliations:** 1 Department of Medical Imaging, Radboud University Medical Center, PO Box 9101, 6500 HB Nijmegen, The Netherlands; 2 Center for Magnetic Resonance Research (CMRR), University of Minnesota, Minneapolis, MN USA; 3 Erwin L. Hahn Institute for Magnetic Resonance Imaging, Essen, Germany

**Keywords:** mpMRI, Prostate cancer, Lymph nodes, Ultra-high-field (UHF) MRI, Metabolic imaging

## Abstract

Multiparametric MRI of the prostate at clinical magnetic field strengths (1.5/3 Tesla) has emerged as a reliable noninvasive imaging modality for identifying clinically significant cancer, enabling selective sampling of high-risk regions with MRI-targeted biopsies, and enabling minimally invasive focal treatment options. With increased sensitivity and spectral resolution, ultra-high-field (UHF) MRI (≥ 7 Tesla) holds the promise of imaging and spectroscopy of the prostate with unprecedented detail. However, exploiting the advantages of ultra-high magnetic field is challenging due to inhomogeneity of the radiofrequency field and high local specific absorption rates, raising local heating in the body as a safety concern. In this work, we review various coil designs and acquisition strategies to overcome these challenges and demonstrate the potential of UHF MRI in anatomical, functional and metabolic imaging of the prostate and pelvic lymph nodes. When difficulties with power deposition of many refocusing pulses are overcome and the full potential of metabolic spectroscopic imaging is used, UHF MR(S)I may aid in a better understanding of the development and progression of local prostate cancer. Together with large field-of-view and low-flip-angle anatomical 3D imaging, 7 T MRI can be used in its full strength to characterize different tumor stages and help explain the onset and spatial distribution of metastatic spread.

## Introduction

MRI of the prostate has emerged as a reliable noninvasive imaging modality for identifying and characterizing focal regions within the prostate. A multiparametric MRI (mpMRI) examination consists of the acquisition of T_2_‐weighted (T_2_w), diffusion‐weighted (DWI) and gadolinium‐based dynamic contrast-enhanced images (DCE), deriving both anatomical and functional information from the prostate. Qualitative and quantitative parameters from such an mpMRI exam correlate with pathological Gleason score and help identify clinically significant cancer [1, 2]. Identification of extra-prostatic extension of the lesion and seminal vesicle invasion is also possible on mpMRI, improving localized disease staging, which is essential for personalized treatment planning [3].

The ability of mpMRI to accurately localize areas suspicious for cancer in the prostate allow for selectively sampling of high-risk regions with MRI-targeted prostate biopsies. In comparison with standard systematic biopsy, guided by ultrasound, prebiopsy MRI followed by MRI-targeted biopsy has the potential to not only improve detection of clinically significant cancer, but also to reduce unnecessary biopsies and over-diagnosis of low-grade, clinically insignificant disease [4]. Active surveillance of low-grade cancer instead of direct therapy is considered a viable management option, as a recognition of the often slow progression of the disease and the importance of maintaining good quality of life [5].

Focal minimally invasive treatment options also take advantage of accurate localization and characterization by MRI for MRI-guidance during the procedures, as well as for the evaluation of treatment success after focal therapy [6]. Currently experimental focal therapies such as high-intensity focused ultrasound, cryoablation and focal laser ablation may become an alternative to radical prostatectomy and high-dose conformal radiotherapy. These focal methods of treatment for patients with organ-confined prostate cancer could reduce adverse events associated with radical treatment whereas retaining at least equivalent cancer control [7]. mpMRI may also play a crucial role in the evaluation of recurrent prostate cancer after focal or any whole gland treatment.

With the development of more personalized treatment options, determining the best treatment strategy for each patient becomes crucial. Whereas localization of a tumor within the prostate is now very well possible with mpMRI [4], assessing its aggressiveness and local and nodal stage still seems difficult, with current clinical mpMRI methodology showing large variation in performance between sites and readers [8]. The use of mpMRI for management of prostate cancer coincided with the introduction of MRI scanners with a magnetic field strength of 3 Tesla in the clinical arena [9]. Using even higher magnetic fields (e.g., 7 T) may bring further benefits in answering clinical questions by allowing imaging of the prostate with unprecedented detail. To date only used in a research setting, the increased magnetic field strength comes with a higher signal-to-noise ratio (SNR) and increased spectral resolution, both of which benefit magnetic resonance spectroscopy studies of the prostate [10]. With increased SNR, existing imaging methods can be improved, but also methods such as phosphorus (^31^P) spectroscopic imaging could become feasible as an imaging biomarker of prostate cancer aggressiveness despite its naturally lower sensitivity [11].

Moving beyond 3 T and making use of the advantages of an ultra-high magnetic field strength poses some challenges [12]. The most significant one being the heterogeneity in the electromagnetic fields in the radiofrequency (RF) range used to excite, refocus and receive the MR signals. The complexity of the transmit magnetic field (B_1_^+^) distortions scale with field strength and the size of the imaging region of interest and can lead to a heterogeneous distribution of flip angles resulting in varying contrast and signal loss throughout the images. In parallel, the heterogeneity in the accompanying complex electric fields can result in high local specific absorption rates (SARs). Furthermore, as susceptibility effects also scale with static field strength (B_0_), inhomogeneities in B_0_ present additional challenges for prostate mpMRI and spectroscopy at 7 T. Finally, the use of ultra-high-field (UHF) body MRI requires custom hardware and software methods, as no vendor currently provides adequate solutions and no existing solutions are approved for clinical translation.

This review will describe the work performed by several research groups to overcome UHF challenges for prostate MR as well as the potential role for MRI/MRS at 7 T in prostate cancer management. We will present an overview of different RF coil designs to facilitate pelvic and prostate imaging at UHF, and we discuss the results of—and outlook for—in vivo measurements to illustrate the potential for a better understanding of prostate cancer and involved lymph nodes in the pelvis with anatomical, functional, and metabolic MRI.

## Radiofrequency coil designs for pelvic MR at 7 T

At 7 T, the inhomogeneity of the RF transmit field combined with increased power requirements and power depositions hinders the use of whole-body RF transmit coils, as is the norm at 3 T. While there is some development on whole-body transmitters [13], local transmit coils which also function as receivers are most practical and are most commonly used for 7 T body imaging. Still the complex B_1_^+^ fields and the greater power depositions at 7 T present challenges in the development and safe use of high-performance local transmit-receiver (transceiver) coil arrays for imaging of the prostate.

Coil designs with multiple independent transmit elements allow for parallel transmission to manipulate the transmit field distribution. These coils are most effectively used when coupled with a system equipped with parallel transmit hardware which has been previously described in the context of UHF body imaging [14]. The possibility to modulate the phase and/or magnitude of each transmit element in an array enables so-called B_1_^+^ shimming. Metzger et al. demonstrated for the first time successful prostate imaging at 7 T with an eight-channel stripline array combined with subject-dependent B_1_^+^ shimming [15]. Using a transceiver surface array coil and altering the input phase of each RF channel based on a relative B_1_ phase map rapidly obtained in vivo for each channel, it was possible to maximize B_1_^+^ phase coherence and thus improve B_1_^+^ homogeneity and transmit efficiency in a region of interest around the prostate. The improved transmit efficiency reduced the required RF power for a given flip angle and reduced local SAR. By doubling the element density by going from an 8-channel to 16-channel stripline array [16], an increase in the expected transmit and parallel imaging performance was achieved at the expense of increased decoupling complexity. SNR increased by 22% for the 16-channel array while lower local and global SAR was exhibited.

As an alternative to the stripline array element, Raaijmakers et al. introduced variations on the dipole for constructing body imaging arrays [17]. Eight single-sided adapted dipole antennas were combined in a surface array aimed at imaging deeply located structures in the body at 7 T, e.g., the prostate. Comparisons to a loop-coil and stripline arrays show that the B_1_^+^ level at the deeper regions is higher for the dipole array with a more symmetrical and uniform B_1_^+^ field distribution. The addition of a tuned inductance in each leg of the dipole in the form of a modified conductor trace (i.e., meanders) leads to what was referred to as the fractionated dipole antenna. RF arrays of the fractionated dipoles lead to improved SAR performance and more homogeneous transmit and receive profiles in the body [18]. At relatively low SAR levels, dipole antennas provide improved B_1_^+^ efficiency and homogeneity at greater depths compared to loop or stripline elements, without the need for subject-specific tuning and matching [18, 19]. Another 8-channel array composed of center-fed microstripline resonators with meanders presented by Orzada et al. [20] also performs well for body imaging at 7 T with a similar quality where patient specific tuning and matching is not required.

As loop coils provide high transmit and receive performance at shallower depths, these coil designs are mostly seen in endorectal coils (ERC). Different configurations were developed, e.g., single-loop [21], two-loop, and microstrip-loop [22–24], either as transceiver or as receive only in combination with a transmit external surface array. In a direct comparison, a two-loop ERC performed best in SAR efficiency, and SNR at 3 cm from the coil, thereby enabling high-quality prostate imaging at 7 T in combination with an external surface array [23]. External transceiver loop-coil arrays have been used to image the heart at 7 T [25]. Loop coils have also been combined with dipole elements in an external transceiver array in work by Erturk et al. [26]. This 16-channel combined loop-dipole transceiver array exploited both the higher transmit and receive performance of the loop elements at shallower depths and improved the performance of the dipole elements at greater depths. The complimentary field patterns of the dipole and loop resonant structures lead to improved performance compared to arrays comprised of loops or dipoles alone.

Somewhat in line with more clinical coil setups, local receive loop-coil elements have also been added at 7 T to increase SNR closer to the body surface and allow larger acceleration factors in parallel imaging. An 8-channel stripline with meander elements array was combined with 24 additional receive-only loop coils for an 8Tx/32Rx setup [27], and also an 8-channel transceiver dipole array was extended with 16 receive-only loop elements, albeit first applied in the upper abdomen [28]. To fully resemble a clinical system with an integrated RF coil, a complete 32-channel parallel transmit system was designed of which the coil elements were placed in the gap between the gradient coil and liner of the patient tunnel [13]. This transmit array had a wide B_1_ transmit field of 50 cm, and could be combined with receive-only loops [29]. Although this setup provides more freedom and comfort to the patient, as only a thin layer of loop coils is needed on top of and below the body, it remains challenging to transmit at high power with well-decoupled distant coil elements.

## Power limits for the body: the specific absorption rate

With increasing magnetic field strength and correspondingly higher RF frequencies, SAR becomes a major issue in the human body. At 7 T and higher, RF field distributions in the torso become largely inhomogeneous, causing local SAR hot-spots inside the body. Using parallel transmit systems, the RF field can be tailored to manage RF inhomogeneity. Still the average RF power needs to be constrained to limit SAR below IEC guidelines [30], with regulatory limits reached much sooner than at 3 T. At UHF, the application of RF over time is generally not limited by whole-body average (global) SAR but rather by local SAR, which cannot be measured directly. Usually, local SAR is numerically estimated using detailed computer-aided models of the human body and of the RF coil with a variety of B_1_ shim settings [11, 16, 18, 26]. Worst-case SAR levels from these simulations lead to channel-specific RF power limits for subsequent in vivo imaging, where these conservative power limits translate into limitations in pulse sequences that traditionally are in need of this RF power (e.g., less slices, longer repetition times or longer RF pulse durations in turbo spin-echo sequences). Subject-specific RF shimming could personalize estimation of locally deposited RF power, and could, therefore, allow more power to be transmitted for individual subjects within SAR safety limits than the general worst-case scenario prescribes. A fast and robust assessment of B_1_^+^ fields in the body of the subject is a first step in this direction [31].

Specific for prostate applications, the extent of local SAR variability between subjects is investigated by Ipek et al. [32]. Four body models were created by segmenting water and fat separated MR images of four volunteers scanned at 1.5 T with the 7 T single-sided dipole antenna coil [17] in place. Simulations found maximum potential local SAR_10g_ levels for 1 W time-averaged accepted power per transmit channel ranging from 4.1 to 7.1 W/kg. These variations show that one model is not sufficient to determine safe power limits. To accommodate the fact that patient specific electromagnetic models are not practical to construct currently, the intersubject variability can be accounted for by assigning a safety factor of 1.7 which is used to scale the estimated SAR determined by the MRI system. After developments in coil design, a newer fractionated dipole antenna [18], was investigated by SAR simulations with the same method but now in 23 body models built from segmented 1.5 T images [33]. For this setup, the maximum local SAR_10g_ for 8 × 1 W input power ranged from 2.6 to 4.6 W/kg. This range still requires a safety margin for intersubject variability of factor 1.78 when using only 1 model in testing of a specific coil design.

Fast subject-specific B_1_^+^ mapping in the pelvis in combination with an alternative way of calculating individualized SAR estimates has been explored for a multi-channel coil array setup [31]. This method used an intermediate offline step to calculate the shims and corresponding power limits taking about 2 min. Ideally, SAR calculations should be individualized to the subject on the table and calculated within a few seconds on the basis of a short preparation scan, altogether automated in one adjustment of the MR system. Meliado et al. aimed for subject-specific local SAR estimates by training a convolutional neural network to estimate a SAR distribution based on a B_1_^+^ map [34]. Training samples were created from the 23 subject-specific body models consisting of synthetic complex B_1_^+^ maps and local SAR distributions. After training, the network took a few milliseconds to predict the accurate local SAR distribution from the presented in vivo B_1_^+^ map. The peak local SAR estimation showed an overestimation error of 15%, reducing the uncertainty in previous SAR assessment methods. This deep learning method allows online image-based subject-specific local SAR assessment, which could dramatically reduce the time for individual system adjustments in a 7 T MR examination.

A different approach to reduce time for system adjustments is the use of a single amplitude and phase shim setting of the transmit channels of a particular transmit coil for all subjects. A fixed RF shim based on the relative phase settings of 12 patients using an 8-channel transmit coil [35] demonstrated that this strategy works but at the expense of B_1_^+^ homogeneity and maximum achievable peak B_1_^+^ in the prostate. Incorporating gradient modulation during the RF pulse in the concept of universal pulses has been embraced for the brain [36] and holds promise for organs in the body as well.

## Anatomical imaging of the prostate: T_2_-weighted imaging

Clinical anatomical imaging of the human prostate predominantly relies on T_2_ contrast, as proton density and T_1_ contrasts are minimal between prostate zones and tumor tissue [37, 38]. T_2_w imaging is the dominant MR series in the overall assessment for abnormalities in the transition zone in the prostate imaging reporting and data system (PI-RADS) [37]. Prostate anatomical images are, therefore, most commonly acquired using a multislice 2D T_2_w turbo spin-echo (TSE) sequence. Due to the high RF power deposition induced by the large number of refocusing pulses used by this sequence, it is restricted in its use at 7 T, currently often translated in the acquisition of fewer slices, prolonged refocusing pulse durations, or longer repetition times at 7 T.

For stripline arrays, initial studies of the feasibility of T_2_w TSE imaging at 7 T in multiple slices or with multiple prostate cancer patients were performed using both an ERC and transceiver external surface stripline array [24, 39]. In work by Metzger et al., the combination of coils was compared to both of them separately [24]. The transceiver external coil (16-channel micro-stripline array) was able to provide a homogeneous B_1_^+^ with uniform contrast across the prostate, while the receive ERC increased the sensitivity near the rectum. The combined coil setup provided the highest SNR and an initial T_2_w image with in plane resolution of 0.5 × 0.5 mm^2^ was acquired. Van den Bergen et al. showed that T_2_-weighted images can be obtained with the eight-element microstrip array within safety guidelines for RF power deposition [39]. Within the SAR limits, five slices of 3 mm could be imaged with a voxel size of 0.8 × 0.8 mm^2^ in a scan duration of 5 min. In the T_2_w images obtained from a single patient, the prostate cancer lesion was visible next to the peripheral and central zone. Although a separate transceiver ERC can deliver a higher peak B_1_^+^ to the prostate, its inhomogeneous field is not suitable for many refocusing pulses in T_2_w TSE, so the first use of this ERC was shown for spectroscopic imaging [21]. A combination of transceiving with both an external coil array and an ERC has not been tested.

As these studies demonstrated the feasibility of T_2_w TSE imaging at 7 T using only a limited number of slices due to SAR restrictions, following work by Maas et al. was aimed to demonstrate full prostate coverage in T_2_w imaging at 7 T [35]. Here, only an external transceiver body array coil was used [20]. After measuring T_2_ relaxation times, the T_2_w-TSE protocol was defined with prolonged excitation and refocusing pulses to reduce the SAR levels. Results from 9 healthy volunteers and 12 prostate cancer patient showed high-quality T_2_w images, acquired in less than 2 min in all subjects with a resolution of 0.75 × 0.75 × 3 mm^3^. Within the set SAR limit (locally 10 W/kg), 28 consecutive slices could be obtained, sufficient for full prostate coverage with 3 mm slices. In the three patients with histopathological proven tumor localization, the tumors were clearly visible on 7 T T_2_w imaging. The quality of the T_2_w images obtained by this method at 7 T was clinically assessed in subsequent work [40]. Three radiologists scored T_2_w images from 17 prostate cancer patients for image quality and the visibility of anatomical structures. Clinically significant cancer lesions were visible both in the peripheral and transition zone, confirming achievable adequate anatomical imaging at 7 T.

In a pilot study to extend the T_2_w imaging to a mpMRI examination with DWI and ^1^H-MRSI, an endorectal receive coil was used in combination with the external array coil for transmission [41]. In 13 patients, with this coil combination, T_2_w imaging was achieved at a spatial resolution of 0.3 × 0.3 × 2 mm^3^. Overall T_2_w image quality was scored as fair (38%) to good or very good (55%), showing sufficient quality for diagnostic purposes at ultra-high resolution at 7 T. Areas of low SNR were encountered in the T_2_w images, resulting possibly from an asymmetrical receive field of the ERC or inhomogeneous B_1_^+^ field resulting in varying flip angles throughout the prostate. Peri-prostatic lipid tissue is not visible on these images, because of opposite gradient polarity of excitation and refocusing pulses with different chemical shift artifacts (different pulse lengths). Muscles, lipids and bones outside the B_1_^+^ shimmed area are subject to low excitation and refocusing flip angles and are outside the receive profile of the ERC, strongly decreasing visibility.

A simplified coil design, getting around the need for parallel transmission and RF shimming, was proposed using loop elements by Rosenkrantz et al. [42]. Within the small ROI of the prostate, uniform flip angles were achieved and an SNR gain of 2.1 was seen versus 3 T imaging, while no comparison was made with other coil configurations. In two patients, 7 T T_2_w imaging demonstrated higher estimated SNR in benign peripheral zone and tumor compared with 3 T, but lower SNR in fat and slight decreases in tumor-to-peripheral zone contrast and homogeneity of the peripheral zone of the prostate.

Dipole coil designs were also put to the test of acquiring T_2_w images of the prostate. For the study of Steensma et al. [43], an 8-element fractionated dipole surface array was combined with a 16-channel receive-only loop-coil array. Compared to 3 T, SNR increased in a range from 1.7 to 2.8 times in the prostate, in a single slice for four volunteers. T_2_w imaging showed improved tumor-to-tissue contrast at 7 T of a single patient, at an improved spatial resolution 0.5 × 0.5 × 2 mm^3^. The same research group also tested the combination of the fractionated dipole surface array and a forward view antenna [44], an external cross-dipole transceive element placed between the legs of a subject against the perineum. This addition led to an increase in SNR of 18% compared to fractionated dipole only. Further variations of a dipole surface array were aimed at reducing local SAR and increasing SAR efficiency [45–47], and although shown successful in simulations or a single volunteer, future measurements in patients are needed to assess the use in vivo at 7 T.

**Fig. 1:**
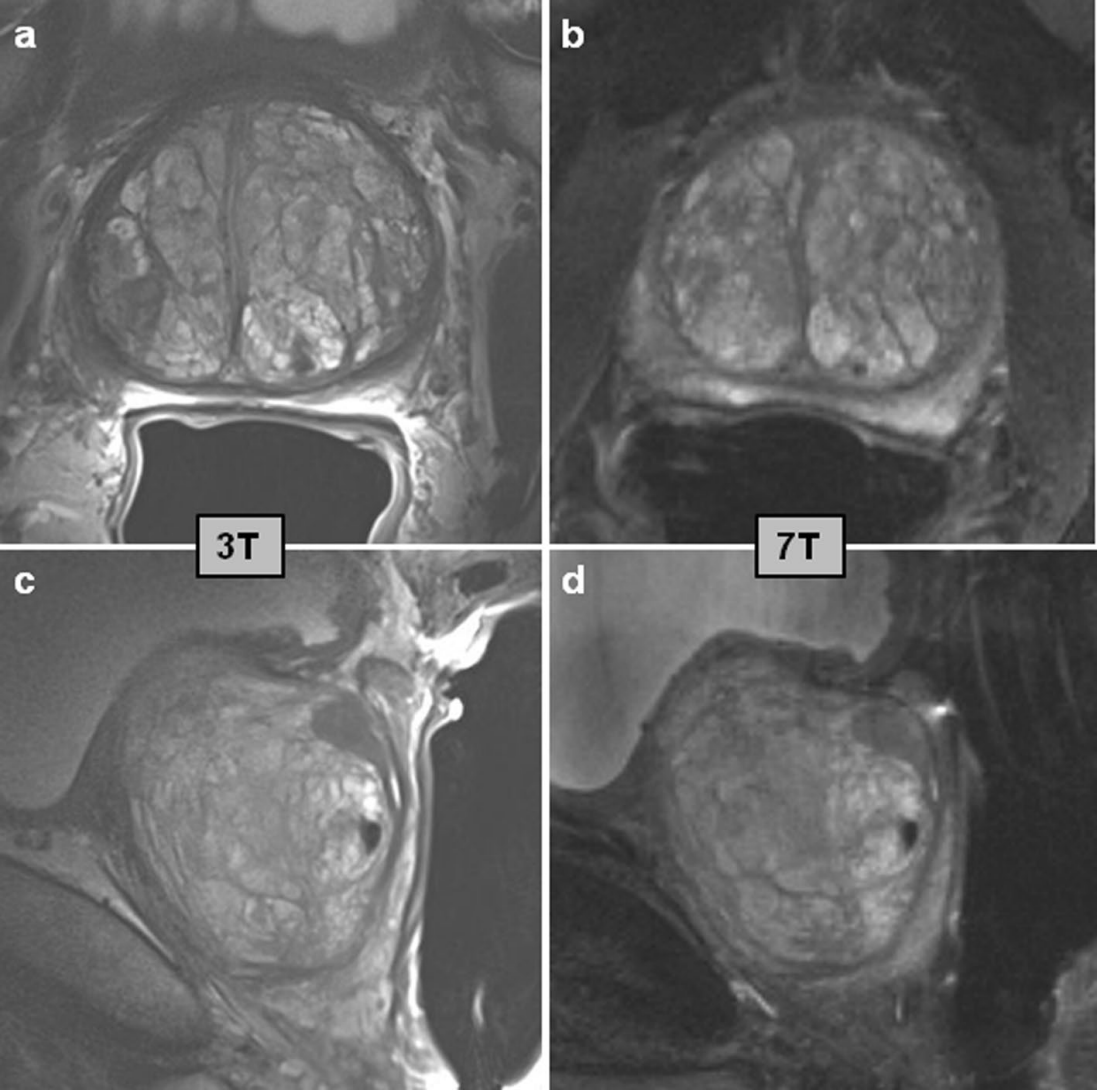
T_2_w imaging of the prostate in two orientations. Axial (**a**, **b**) and sagittal (**c**, **d**) slices of the prostate of a patient with prostate cancer at 3 T (**a**, **c**) and at 7 T (**b**, **d**). The patient had a Gleason 3 + 4 lesion in the right midgland peripheral zone. The 3 T data was acquired with an endorectal coil (Medrad^®^, Pittsburgh, PA, US) at a spatial resolution of 0.4 × 0.4 × 3.0 mm (TE 101 ms, acquisition time 4:21 min for each orientation). 7 T data was acquired as in [35] with an external 8-channel transceiver coil after phase shimming at a spatial resolution of 0.75 × 0.75 × 3.0 mm (TE 71 ms, acquisition time 1:30 min for each orientation)

The studies described here show the feasibility of T_2_w imaging of the prostate at 7 T with a significant increase in SNR. This advantage is heavily counteracted by SAR limitations and available transmit power, resulting in limitations for in plane resolutions, total number of slices, or repetition times. In a comparison to clinical T_2_w MRI at 3 T (Fig. 1), the increased sensitivity at 7 T for T_2_w TSE has not yet been fully exploited, which can be addressed through the continued development of optimal receiver arrays, RF management strategies and acquisition techniques. For example, new acquisition strategies providing more flexibility at 7 T may include 3D gradient echo imaging at high spatial resolution using magnetization preparation pulses. When prepared with T_2_w modules [48], low-flip-angle excitations with gradient echo readouts could provide 3D high isotropic resolution with low SAR demands.

## MRI of restrictions in water motion: diffusion-weighted imaging

In PI-RADS, DWI is the dominant mpMRI series for assessing peripheral zone lesions. Scores are assigned based on the lesions appearance on apparent diffusion coefficient (ADC) maps and DWI images at (calculated) high b values (≥ 1400 s/mm^2^) [37]. Increasing the magnetic field strength from clinical 3 T to experimental 7 T comes with an increase in magnetic susceptibility differences within tissue or at tissue-air interfaces. This makes DWI of the prostate at 7 T challenging.

**Fig. 2:**
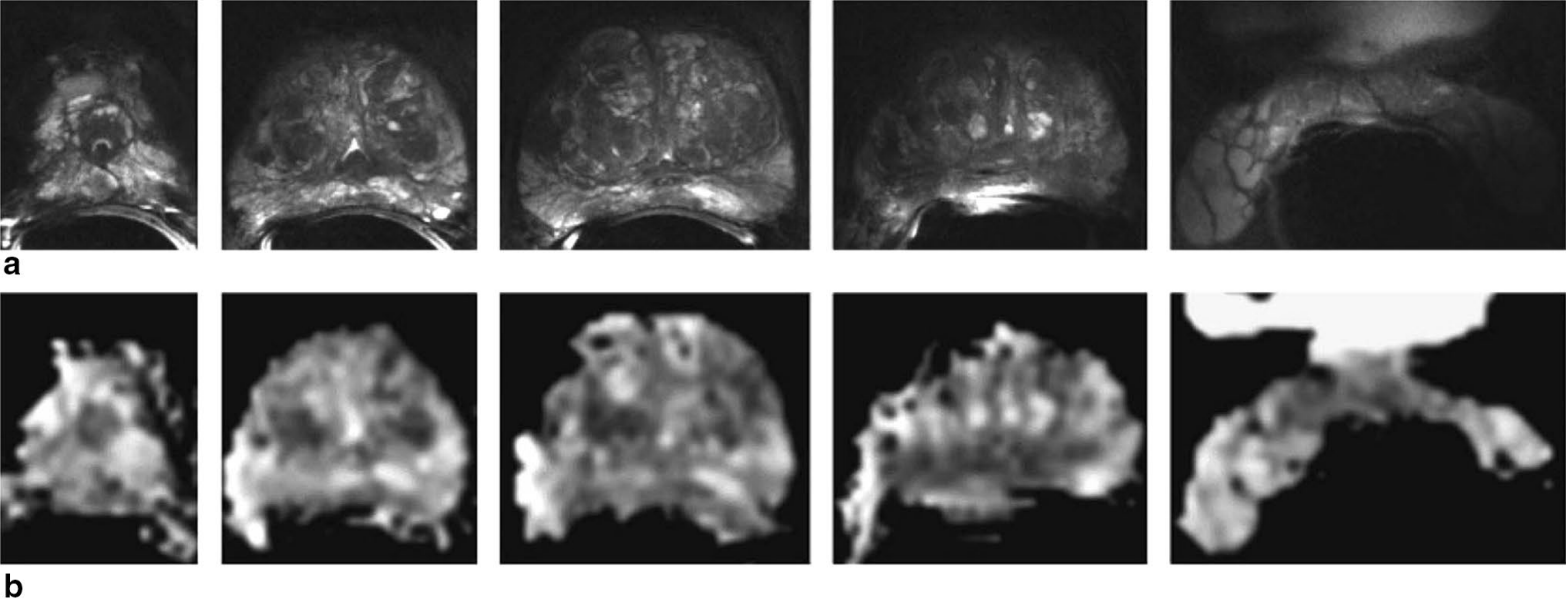
MRI with an ERC at 7 T of the prostate of a 62-year-old patient with prostate cancer (Gleason score 3 + 3). **a** Five transversal T_2_w turbo spin-echo images from a series of 23 slices from apex to seminal vesicles, resolution 0.3 × 0.3 × 2.0 mm^3^. **b** Transversal ADC maps at corresponding locations (5 images from 23 slices), resolution 1.75 × 1.75 × 2.0 mm^3^. Reprinted with permission from Lagemaat et al. [41]

In the mpMRI acquisition by Lagemaat et al. [41], DWI was acquired at 7 T with an external transmit array and endorectal receive coil using an echo-planar imaging (EPI) sequence with b-values of 0, 100, 400 and 800 s/mm^2^. Apparent diffusion coefficient maps and b1400 images were calculated using a mono-exponential decay model. Results showed that DWI at a resolution of 1.75 × 1.75 × 2 mm (or 1.4 × 1.4 × 2 mm in two patients) was possible at 7 T at a rating of good to very good in 11 out of 12 patients. Anatomical structures, as well as the cancer lesions, were visible on the ADC maps since geometric distortion of the prostate with respect to T_2_w imaging was very small (Fig. 2). DWI images were affected by a ghosting artifact of the rectal wall in the readout direction limiting the robustness of this technique.

Improved DWI performance at 7 T can be obtained by adopting methods currently used clinically at 3 T but have yet to be implemented on UHF systems. Along with parallel imaging, reducing the imaging FOV in the phase encode direction through the use of 2D selective excitation pulses reduces susceptibility artifacts and minimizes SNR with a shortened gradient echo train length and echo times. A similar strategy was implemented where a reduced FOV in the anterior–posterior (AP) direction was facilitated using AP regional saturation bands when using a body array for transmit and a combined endorectal-body array for receive [49]. Alternatively, similar to T_2_w preparation modules, diffusion-preparation modules could be combined with low-flip-angle excitations and gradient echo readouts, although no examples at 7 T currently exist.

## Dynamic contrast-enhanced MRI of the prostate

While dynamic contrast-enhanced MRI’s role in the overall assessment of prostate lesions in PI-RADS version 2 is limited, it remains a continued component of the clinical mpMRI exam for prostate cancer detection [37]. The benefit of 7 T in DCE MRI would be an increase in spatial and/or temporal resolution for improved pharmacokinetic modeling or qualitative assessment of contrast enhancement curves. To date, only limited DCE MRI examinations have been performed in prostate cancer patients at 7 T [49]. To perform these studies, it was important to consider the increased R_2_* (1/T_2_*) relaxivity of Gadolinium based contrast agents at 7 T. While the R_1_ (1/T_1_) relaxivity, which gives the desired enhancement for DCE MRI, remains relatively constant with increasing field strength, the R_2_* relaxivity was shown to increase nearly fourfold at 7 T compared to 3 T [50] thus dominating the observed signal changes especially in the blood where the concentrations are the highest. To avoid biasing the interpretations of these data, either the time-varying T_2_* needs to be calculated and corrected for through a multi-TE acquisition, or ultra-short TE acquisitions can be used to mitigate the T_2_* effects [14].

## Assessment of metabolite ratios within the prostate with ^1^H-MRSI

Proton MRSI of the human prostate has been shown useful at 3 T as healthy and cancerous prostate tissue regions show differences in choline and citrate signal intensities [51]. Different ratios of choline, polyamines, creatine and citrate in combination with anatomical T_2_w MR imaging can identify and localize prostate cancer. Moreover, these ratios can be used to discriminate between different Gleason Grade groups [52], indicating a role for ^1^H-MRSI in characterizing prostate cancer. The demand for specific expertise and relatively long acquisition times have prevented this technique to be included in routine mpMRI examinations, as described in the latest PI-RADS version [37]. Since ^1^H-MRSI could benefit most from the higher SNR and spatial resolution at 7 T, this was the first MRI technique to be tested for prostate examinations at 7 T. To confirm the potential of prostate ^1^H-MRSI, challenges posed by the B_1_^+^ inhomogeneities and increased chemical shift artifacts had to be overcome for this technique at 7 T.

First, dedicated coil designs for ^1^H-MRSI at 7 T were developed. The use of an ERC, if used for RF transmission, can provide high but non-uniform peak B_1_^+^ fields in the prostate with relatively low RF power [21, 53]. The second challenge, the chemical shift displacement artifact, causes artefactual spatial variations of the citrate to choline ratios and results in contamination of spectra in the volume of interest (VOI) with signals from outside of this VOI. Overcoming both challenges, even in the presence of the large B_1_^+^ fields generated by the transmit ERC, was done using adiabatic RF pulses [21]. ^1^H-MRSI sequences developed for 7 T are either semi- of fully adiabatic (called semi-LASER or LASER) [21, 54]. The shortest achievable echo time for a semi-LASER sequence including dual-frequency-selective pulses for water and lipid suppression and with an optimal shape of the strongly coupled citrate resonances was 56 ms, which minimizes signal degradation due to T_2_ relaxation [21]. The addition of chemical shift selective pulses to the semi-LASER sequence to refocus the spermine spins allows obtaining maximum signal of all metabolite of interest in the prostate, albeit at a longer echo time [55].

These dedicated coil designs and pulse sequences were tested in multiple patients. In a study by Luttje et al., five patients with prostate cancer underwent an MRI examination at 7 T with the use of a ^1^H/^31^P transmit and receive ERC [53]. Two-dimensional ^1^H-MRSI was acquired with a composite adiabatic excitation and refocusing pulse [54], taking advantage of the spatial excitation properties of the ERC in the right-left direction of the prostate. At an acquired voxel size of 5 × 5 × 5 mm^3^ and an acquisition time of 7.46 min, the in vivo spectra showed well-resolved peaks for choline, polyamines, creatine and citrate. The spectra from a tumor region showed an increased total choline peak and decreased polyamine and citrate signals. The sensitivity of the ERC decreased with distance from the coil, resulting in limited coverage in the anterior direction of the prostate.

To allow 3D coverage of the entire prostate, the combination of a body array transmit coil and ERC for reception was tested by Lagemaat et al. [41]. This combination of a transmit surface array coil and receive ERC was able to provide a homogeneous B_1_^+^ field in the prostate with detection at high SNR [41, 56]. Patients were subjected to an mpMRI examination, consisting of T_2_w, DWI, and ^1^H-MRSI. As the homogeneous B_1_^+^ field obviated the need for the LASER sequences, less RF power demanding pulses could be used. Dedicated spectral-spatial refocusing pulses for both spectral and volume selection were developed at 7 T, as a more SAR efficient alternative to the semi-LASER sequence [57]. As these pulses perform frequency selection of the ppm-range of interest next to volume selection, the need for additional water and lipid suppression pulses was eliminated. From 12 patients with prostate cancer, 3D ^1^H-MRSI was obtained with this method (real voxel size: 0.6 cm^3^ in 7:16 min) [41]. The MRSI quality was rated fair to good in 56% of the acquisitions. Again, signal levels were decreased in spectra more distant from the ERC. Spectra from tumor tissue showed decreased citrate levels compared to the total spermine and choline peak in one patient and an increased choline level compared to healthy prostate tissue in another. Lipid contamination influenced the spectral quality, hindering detection of citrate and indicative of remaining inhomogeneities in the B_1_^+^ field. Effort has also been put into the development of parallel transmit-designed adiabatic spectral-spatial refocusing pulses simultaneously providing both spatial localization and signal suppression insensitive to B0 variations [58].

In a 7 T mpMRI feasibility study by Philips et al. [56], using a ^31^P/^1^H ERC with an external ^1^H transceiver body array and spectral-spatial refocusing pulses, ^1^H-MRSI spectra were acquired from three patients. Adequate SNR was seen from apex to base of the prostate resulting in spectra with well distinguishable choline and spermine signals, while citrate in some cases suffered from lipid contamination. Close to the ERC and in the seminal vesicles, the spectral map shows wider peaks because of local B_0_ field inhomogeneities. With a corrected voxel size of 1.4 cc, in a patient with Gleason score 4 + 4, an elevated total choline peak was found compared to the contralateral side.

While the studies above show the feasibility of ^1^H-MRSI in prostate cancer at 7 T, the potential benefit of spectral-spatial pulses for assessing prostate cancer must be investigated in a larger patient cohort with tumors varying in Gleason scores. Spatial resolution can be improved compared to 3 T due to the high sensitivity at 7 T, which would reduce partial volume effects causing voxels to better represent tumor tissue. Further reductions in voxel size will improve the separate detection of total choline and spermine because this will improve intravoxel B_0_ homogeneity, narrowing the linewidths. Ratios including the total choline could then serve to characterize prostate cancer and remove ERC coil profile effects [57]. As the spectral-spatial pulses result in high spermine signals in the ^1^H spectra [53, 56], the value of these spermine signals for prostate cancer characterization should be investigated further, as biopsy studies have shown a significant decrease in spermine concentration with increasing tumor grade [59].

## Metabolic imaging within the prostate with ^31^P MRSI

At a magnetic field strength of 7 T, the higher signal-to-noise ratio and increased spectral resolution creates the opportunity to develop ^31^P spectroscopic imaging of the prostate [10]. Phosphorus spectroscopy enables detection of metabolites with phosphorus atoms, e.g., phosphocreatine (PCr), inorganic phosphate (Pi), the three phosphate groups of adenosine triphosphate (ATP), phosphomonoesters (PME), and phosphodiesters (PDE). The increased chemical shift difference between signals, compared to 3 T, might enable detection of individual resonances of PME, phosphoethanolamine (PE) and phosphocholine (PC), and PDE, i.e., glycerophosphoethanolamine (GPE) and glycerophosphocholine (GPC) [10]. As in vitro studies have shown a significant difference in PE and GPE between cancer and benign prostatic hyperplasia [60] and that ^31^P profiles discriminate between low-grade and high-grade cancer [61], in vivo ^31^P MRSI of the prostate could improve the assessment of prostate cancer aggressiveness [11]. Together with the increased SNR, it could be possible to perform localized 3D ^31^P MRSI at 7 T at a relevant spatial resolution in a clinically acceptable measurement time.

One of the first studies to test the feasibility of ^31^P MRSI at 7 T in the prostate was performed by Kobus et al. [11]. A ^31^P endorectal coil was developed and combined with an eight-channel ^1^H body array coil to couple metabolic and anatomical information. The combination was submitted to an extensive safety validation, demonstrating that the presence of the ^31^P endorectal coil had no influence on the SAR levels and temperature distribution of the external eight-channel ^1^H array coil. The maximum time-averaged input power of the ^31^P endorectal coil was determined and used for the first in vivo tests. In a healthy volunteer, using adiabatic excitation, 3D ^31^P MR spectroscopic imaging produced high-quality spectra from the entire prostate in 18 min with a spatial resolution of 4 cm^3^.

Subsequently, this setup of combined ^31^P transceiver endorectal and eight-channel ^1^H transceiver body coil was tested in an explorative study with 15 patients with biopsy-proven prostate cancer by Lagemaat et al. [62]. The signals of PE and PC were well resolved in most ^31^P spectra in the prostate, and ^31^P MRSI at 7 T resulted in distinct features of phospholipid metabolites in the prostate gland and its surrounding structures. The two patients with high Gleason score tumors (GS: 5 + 4 and 4 + 5) presented with high PC and GPC levels. Although these ^31^P PC and GPC levels may be related to prostate cancer aggressiveness, generally, differences in metabolite ratios between prostate cancer and normal-appearing tissue were not observed possibly because of partial volume effects of small tumor foci in large MRSI voxels.

In parallel work by the same group, the ^31^P MRSI protocol was optimized for the human prostate at 7 T by the evaluation of T_1_ relaxation times and the Nuclear Overhauser Effect (NOE) of phosphorus-containing metabolites in 12 patients [63]. T_1_ relaxation times of ^31^P metabolites in the human prostate at 7 T varied between 3.0 and 8.3 s. The relatively long T_1_ relaxation times of ^31^P metabolites, as well as reasonable limitations in total acquisition time, require a tradeoff between TR and accompanying flip angle and matrix size. Sensitivity of ^31^P MRSI of the prostate at 7 T may be increased by irradiation of water protons, giving rise to NOE enhancement. Positive NOE enhancements were measured for most metabolites, which can improve fitting accuracy, but the variability in NOE results requires further investigation. With a strongly reduced ^31^P flip angle (≤ 45°), a high-quality ^31^P MRSI dataset with optimal SNR per unit time can be obtained within 15 min.

A different coil setup for prostate ^31^P MRSI was developed and tested by Luttje et al. [53], with the aim of acquiring both ^1^H and ^31^P MRSI of the human prostate at 7 T in one scan session. Therefore, a two-element ^1^H/^31^P transmit and receive ERC was designed. As the endorectal transceiver provides high SNR and strong B_1_ fields, detection of the phospholipid metabolites was feasible. Their preliminary in vivo prostate results indicated lower levels of PE in the tumor areas compared to healthy prostate tissue.

**Fig. 3:**
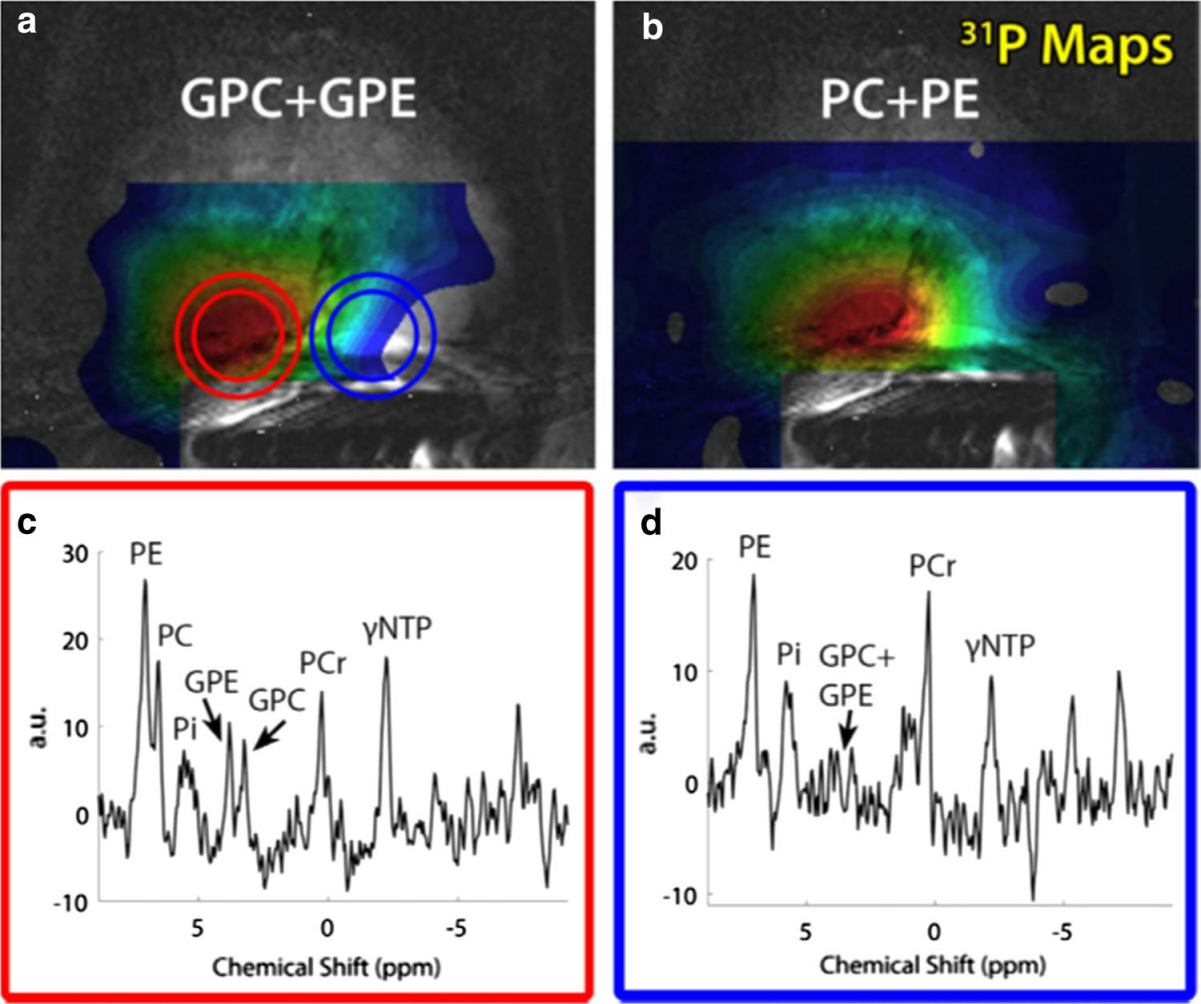
GPC + GPE and PC + PE, metabolite maps of a 67-year-old patient with metastatic Gleason 4 + 4 prostate cancer (**a**, **b**) overlaid over a T_2_w image. ^31^P (**c**, **d**) spectra are shown of the cancer lesion (red circle) as well as a location within healthy tissue (blue circle). Adapted from Philips et al. [56]

As T_2_w imaging of the prostate by a transceiver ERC suffers from B_1_ inhomogeneities, the combination of an ERC with a ^1^H external coil array would be optimal for functional and anatomical imaging. In a study by Philips et al., this strategy was implemented by combining an ERC with a ^31^P transceiver loop-coil and ^1^H receive (Rx) asymmetric microstrip (^31^P/^1^H ERC) with an external ^1^H transceiver body array coil [56]. After extensive safety testing, the total coil combination allowed acquisition of a multinuclear mpMRI protocol, consisting of high-resolution T_2_w imaging and DWI without artifacts and the assessment of ^31^P and ^1^H metabolites of the prostate in three patients with histology proven prostate cancer. In one patient with high-grade prostate cancer (Gleason grade 4 + 4), GPC, GPE, PE and PC levels were identified and elevated in the cancer lesion compared to the healthy contralateral side of the prostate (Fig. 3). In a larger patient population, this combined setup could provide insight in the changes in total choline metabolism in prostate cancer.

The most optimal coil configuration with respect to B_1_^+^ homogeneity and highest SNR for signal reception would be to separate transmit and receive for both nuclei with a body array capable of transmitting on both the ^1^H and ^31^P frequencies and an endorectal coil to receive the signals from both nuclei.

## Staging lymph nodes at ultra-high magnetic field

Next to imaging and characterization of localized prostate cancer, there is also a potential role of 7 T MRI for the identification of lymph node metastases. There is a great clinical need for precise, reliable and noninvasive N-staging in patients with prostate cancer. The excellent soft-tissue contrast of MRI enables visualization of lymph nodes within the surrounding lipid tissue. Using lipid suppression or water excitation, the contrast between the water containing lymph nodes and lipid tissue can be increased [64]. UHF MRI provides an increased spatial resolution compared to 3 T, which enables the visualization of lymph nodes even smaller than 5 mm. With the large chemical shift dispersion between water and lipids at 7 T, selective excitation of either of the two with short simple rectangular pulses becomes an attractive option. This allows very efficient imaging by short excitations in the order of 1 ms and receiving multiple gradient echoes, using approximately 60% of the repetition time to actually acquire MR signal.

In the work by Philips et al. [65], these advantages of 7 T MRI were used in a multigradient echo sequence combined with TIAMO acquisition. Using a 3D water-selective multigradient echo sequence enabled the quantification of the signal decay of the lymph nodes with its corresponding T_2_* relaxation time. As a consequence, images could be computed at any echo time using all gradient echo signals. This method of high-resolution 3D isotropic pelvic imaging over a large field of view performed robustly in six healthy volunteers with a low SAR. Pelvic lymph nodes where found with a size down to 1.5 mm short axis and a mean short and long axis of 2.2 ± 0.1 and 3.7 ± 0.2 mm, respectively. In another set of 11 young healthy volunteers, 69% of the total amount of 564 pelvic lymph nodes found on the water-selective computed TE images were 2 mm or smaller (mean diameter: 2.3 mm, range 1–7 mm) [66].

Next to small-sized lymph nodes, the 7 T MRI would be able to pick up lymph node morphology in patients, such as heterogeneous signal intensity or irregular border and shape. However, morphological criteria cannot safely differentiate metastatic from nonmetastatic lymph nodes. Using a functional MRI contrast like ferumoxtran-10, currently only available for use in clinical trials, one can discriminate between normal lymphatic tissue that has taken up the ultrasmall superparamagnetic particles of iron oxide (USPIO) and metastatic lymph nodes that have not taken up the particles and preserve high MRI signal on T_2_*-weighted MRI pulse sequences [64].

**Fig. 4:**
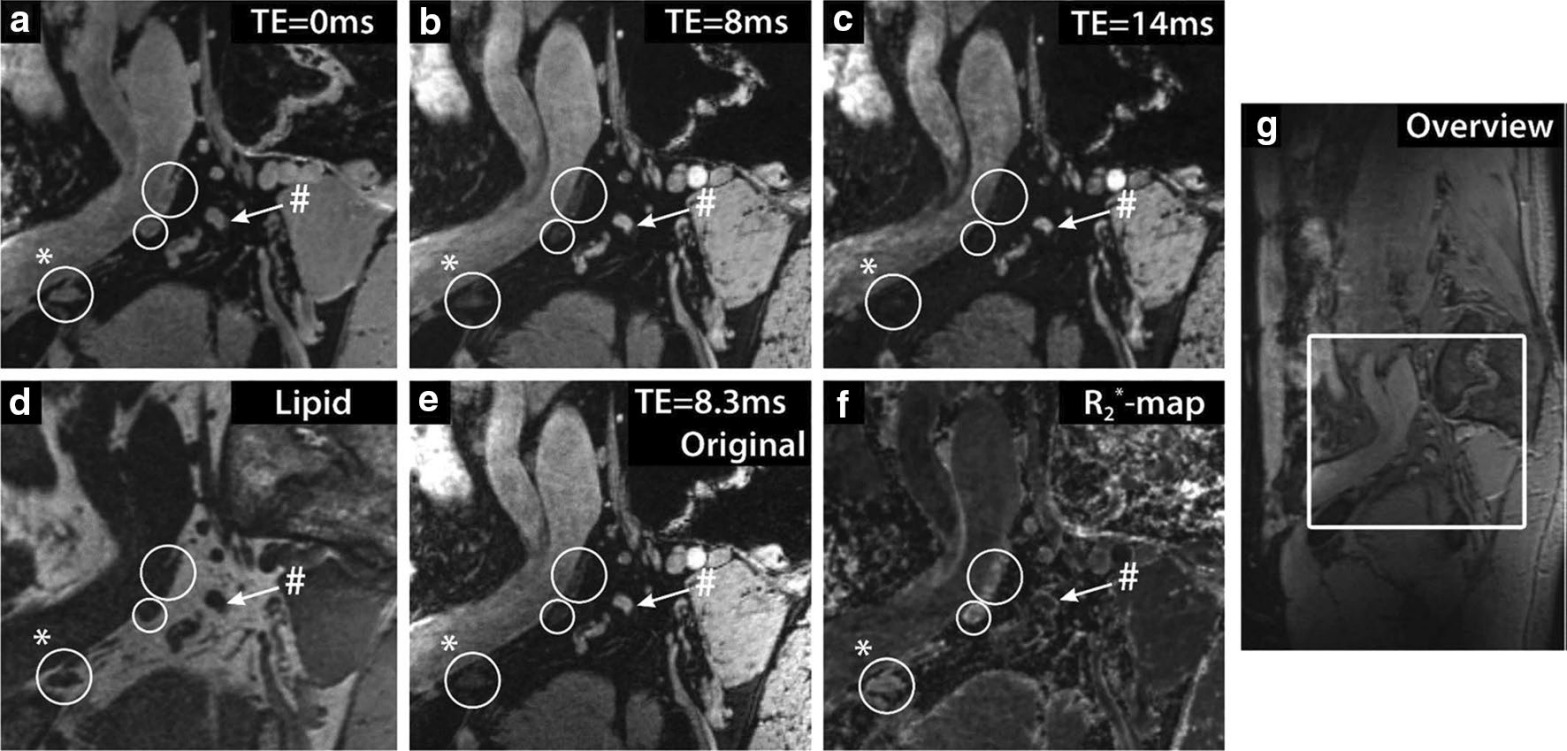
The effect of USPIO nanoparticles at 7 T in the lymph nodes of a 54-year old patient with prostate cancer metastases. **a**–**c** Sagittal images of the same location at different computed echo times (TEs). **d** Lipid-selective image. **e** The original TE = 8.3 ms image from the multigradient echo water-selective imaging. **f** Map of fitted R_2_* relaxation rates. **g** Sagittal overview image with inset of location of images **a**–**f**. Three lymph nodes accumulated USPIO particles and rapidly lost MR signal intensity (white circles), while one suspicious lymph node without USPIOs retained MR signal intensity with increasing TE (white arrow). The lymph node marked with (#) showed a slow signal decay, with an R_2_* value of 80 ± 6 s^−1^, whereas the lymph node marked with (*) showed fast signal decay, with an R_2_* value of 247 ± 25 s^−1^. Reprinted with permission from Scheenen et al. [64]

Performing the same multigradient echo sequence now after USPIO infusion [67], separate scans with either water or lipid excitation provide an anatomical overview while the iron-sensitive signal intensity discriminates between nodes with and without USPIO accumulation. In three patients with rectum cancer and three patients with prostate cancer lymph nodes were identified on computed TE = 0 ms images from water-selective scans in combination with images from lipid-selective scans (Fig. 4). A range of 3–48 lymph nodes without USPIO signal decay was found per patient. This study showed the technical feasibility of USPIO-enhanced MRI at 7 T, enabling the detection of USPIO uptake in normal-sized lymph nodes by the high SNR and unprecedented spatial resolution. The effect of USPIO-enhanced MRI on nodal staging has to be studied as no systematic follow-up or node-to-node pathological data is yet available for prostate cancer. A study by Stijns et al. [68], regarding patients with rectal cancer, revealed challenges to overcome to perform accurate node-to-node matching from clinical USPIO-enhanced MRI to histopathology.

## Prostate imaging at 10.5 T

The exploration of UHF imaging in the human body extends to 10.5 T where a recent study demonstrated the possibility of imaging targets throughout the torso, including the prostate [69]. In fact, the focus of the first human imaging study ever conducted on the 10.5 T whole-body MRI scanner was the prostate of a healthy male volunteer. At 10.5 T, the proton resonance frequency is 447 MHz with an approximate wavelength of 7 cm in the human body resulting in rapidly varying complex field patterns that need to be managed. Using a 10 channel fractionated dipole as a transceiver [70], subject-dependent RF shimming was performed to mitigate the destructive interferences. Again, the main goal is to generate a sufficiently homogeneous and efficient B_1_^+^ in the prostate to perform the sequences and obtain the contrasts typical of an mpMRI exam including T_1_w, T_2_w and DWI acquisitions.

**Fig. 5:**
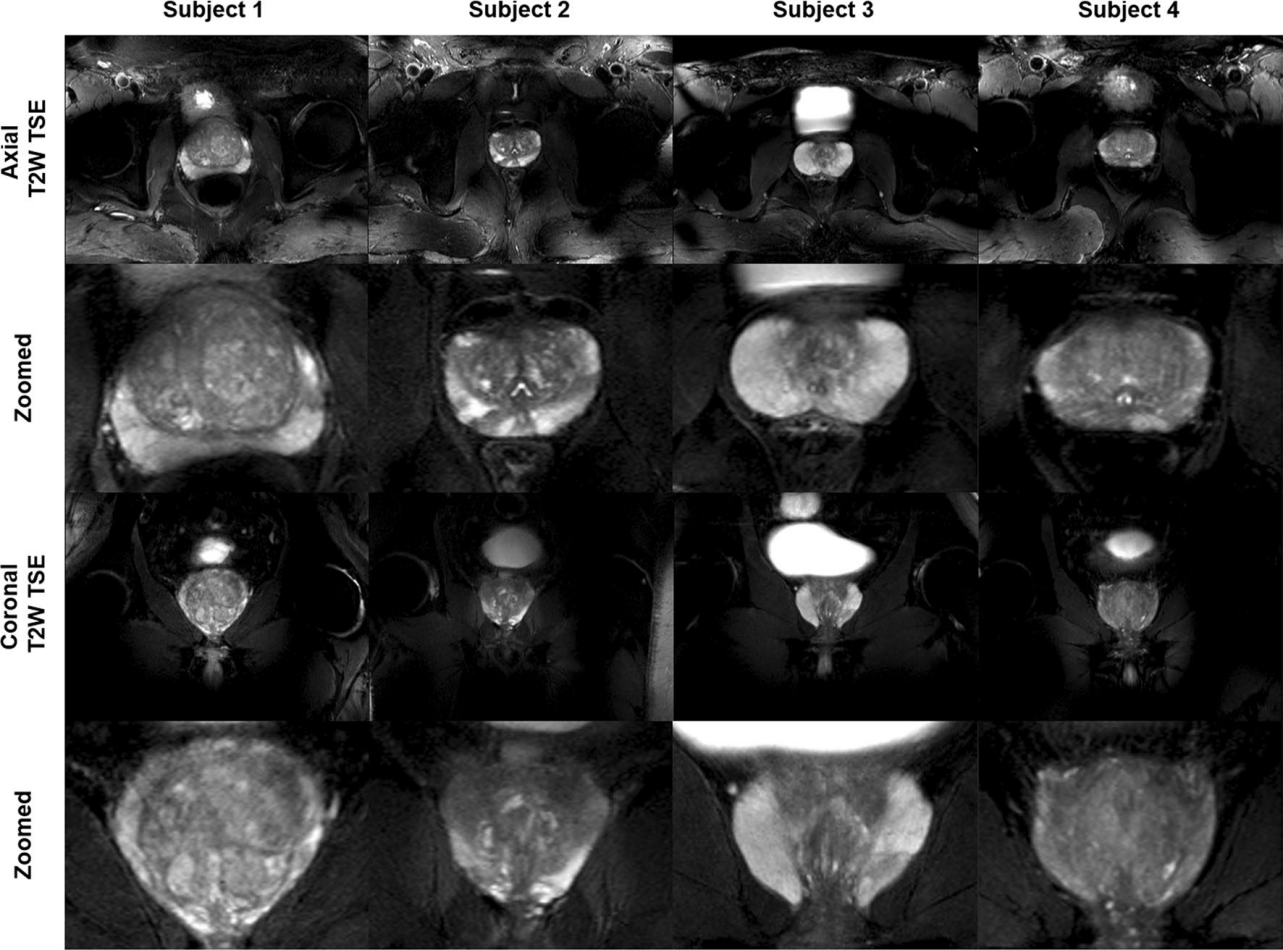
Full FOV and zoomed-in versions of axial and coronal T_2_w TSE images of prostates from 4 subjects. Images demonstrate excellent contrast and SNR achieved by phase-only RF shimming. Although providing high peak B_1_^+^ and reasonable homogeneity over the prostate, the local RF shim resulted in unmanaged RF fields outside the immediate region of the targeted anatomy that caused spatially varying destructive interference patterns as observed in the unzoomed images. Reprinted with permission from He et al. [69]

In contrast to prostate studies at 7 T, an RF shimming strategy focusing solely on RF efficiency [15] was not always adequate at 10.5 T. Depending on body geometry and prostate size, a shim optimized for a tradeoff between the two conflicting requirements of homogeneity and efficiency was required to obtain acceptable image quality [71]. As evident from these results and by the contrasts demonstrated in axial and coronal T_2_w acquisitions acquired across four subjects (Fig. 5), these RF management strategies provided a sufficiently homogeneous and efficient B_1_^+^ to perform prostate imaging in all volunteers. The average B_1_^+^ in prostate across all subjects was 10.2 ± 1.9 μT, and the average coefficient of variations was 13.0 ± 2.4%. Multiparametric mappings in prostate including T_1_, T_2_, ADC and calculated DWI acquired in a subset also demonstrated the feasibility of quantitative imaging at 10.5 T [69].

While standard strategies to minimize power deposition were employed at 10.5 T, such as reduced-flip-angle echo trains for TSE and prolonged repetition times, local SAR limited the number of slices to ≤ 11 of 3 mm while abiding by safety guidelines. This is roughly half that is needed in clinical studies of the prostate to properly cover this anatomy [35]. RF pulses optimized with SAR constraints can be employed to decrease the local peak SAR levels as can RF coil geometries with improved SAR efficiencies to support more time efficient imaging and coverage at 10.5 T [72].

## Conclusion

Significant effort has been invested in addressing the technical challenges of UHF imaging of the lower abdomen, specifically the prostate and its surrounding tissue. The culmination of this work clearly demonstrates that multinuclear multiparametric MR(S)I of the prostate at UHF is feasible. With the exploitation of higher spatial resolutions in imaging and in spectroscopy, mpMRI at UHF can yield improved delineation of prostate anatomy and metabolic imaging within the tumor as a biomarker for an assessment of prostate cancer aggressiveness. For clinical detection of primary prostate cancer, a fast, readily available and robust MRI acquisition is needed; current 3 T protocols fulfill these demands. The potential role of prostate imaging at 7 T could be in achieving a better understanding of the development and progression of local prostate cancer, the onset and spatial distribution of metastatic spread, and in characterization of different tumor stages and recurrent disease.
